# Lactation milk yield prediction in primiparous cows on a farm using the seasonal auto-regressive integrated moving average model, nonlinear autoregressive exogenous artificial neural networks and Wood’s model

**DOI:** 10.5713/ajas.19.0939

**Published:** 2020-04-12

**Authors:** Wilhelm Grzesiak, Daniel Zaborski, Iwona Szatkowska, Katarzyna Królaczyk

**Affiliations:** 1 Department of Ruminants Science, West Pomeranian University of Technology, 71-270 Szczecin, Poland; 2 Department of Animal Anatomy and Zoology, West Pomeranian University of Technology, 71-466 Szczecin, Poland

**Keywords:** Prediction, Heifer, Lactation Curve, Milk Yield, Neural Networks, Statistical Methods

## Abstract

**Objective:**

The aim of the present study was to compare the effectiveness of three approaches (the seasonal auto-regressive integrated moving average [SARIMA] model, the nonlinear autoregressive exogenous [NARX] artificial neural networks and Wood’s model) to the prediction of milk yield during lactation.

**Methods:**

The dataset comprised monthly test-day records from 965 Polish Holstein-Friesian Black-and-White primiparous cows. The milk yields from cows in their first lactation (from 5 to 305 days in milk) were used. Each lactation was divided into ten lactation stages of approximately 30 days. Two age groups and four calving seasons were distinguished. The records collected between 2009 and 2015 were used for model fitting and those from 2016 for the verification of predictive performance.

**Results:**

No significant differences between the predicted and the real values were found. The predictions generated by SARIMA were slightly more accurate, although they did not differ significantly from those produced by the NARX and Wood’s models. SARIMA had a slightly better performance, especially in the initial periods, whereas the NARX and Wood’s models in the later ones.

**Conclusion:**

The use of SARIMA was more time-consuming than that of NARX and Wood’s model. The application of the SARIMA, NARX and Wood’s models (after their implementation in a user-friendly software) may allow farmers to estimate milk yield of cows that begin production for the first time.

## INTRODUCTION

Information on future milk yield performance (even if only approximate) provides premises for making rational decisions and stimulates actions aimed at the realization of favorable prediction (e.g. appropriate animal care). Daily or weekly milk yield prediction allows early identification of low-producing animals, which can subsequently be culled. Perturbations in milk production may indicate health [1,2] or reproductive [3] problems. Farmers can predict milk production level at successive lactation stages based on lactation curve shape [4]. Production costs, expected profits and the economic value of a cow or cows can be estimated based on current milk prices. They are also used for making decisions about subsequent artificial inseminations of such cows, their further utilization and herd replacement. Consequently, the policy of advance planning can be implemented.

Therefore, it seems understandable that the possibility of predicting lactation curve shape of cows is a highly beneficial endeavor, especially for primiparous cows, whose real production performance is unknown despite the previous breeding value estimation. The recording of milk yield in young cows from the previous production periods on a farm is the basis for generating predictions that may be compared with the real values achieved by the next generations of primiparae. Thus, it enables the verification of selection efficiency over successive years.

One of the most popular methods of milk yield prediction is a regression model, especially Wood’s model, but also the so-called artificial intelligence methods (including artificial neural networks) or time series prediction models. Wood’s model, as a mathematical equation describing the relationship between milk yield and lactation duration is quite frequently applied to milk yield prediction in cows [5–7] and often serves as a good reference model for comparison with other prediction models. This model includes parameters associated with milk yield at peak lactation, the stage of milk yield increase and its decrease after the lactation peak.

Another method of lactational milk yield prediction is the seasonal auto-regressive integrated moving average (SARIMA) model, which is based on the auto-regressive integrated moving average (ARIMA) model, whose theoretical basis was developed by Box and Jenkins in 1976 [8]. Assuming that lactation is characterized by certain homogeneity (i.e. excluding the local level and trend, any part of the lactational yield series is very similar in its shape to every other part), the difference among them is a stationary, mixed autoregressive and moving-average process. The SARIMA models are quite frequently used for different prediction tasks, including agricultural ones such as annual milk production prediction in India [9,10], lactation yield prediction in sheep based on test-day records [11], the prediction of weekly and lactational milk yield in Egyptian cows [12] or weekly milk yield in Spanish goats [13].

The next prediction method based on machine-learning, i.e. the recognition of some characteristic patterns of a given phenomenon through the learning process, is a nonlinear autoregressive exogenous (NARX) artificial neural network applied in various domains, including animal breeding, e.g. milk yield prediction in cows during lactation [14–16].

Consequently, the aim of the present study was to verify the usefulness of unconventional SARIMA and NARX methods for the prediction of lactational milk yield in primiparous cows kept on a medium-sized farm and to compare their predictive performance with the reference Wood’s model.

## MATERIALS AND METHODS

Since the present study involved only the analysis of production records routinely collected on the farms, the approval from the Local Ethics Committee on Animal Research was not necessary. The dataset comprised monthly test-day records from primiparous Polish Holstein-Friesian Black-and-White cows kept on one of the dairy farms located in the West Pomeranian Province. The A4 method was used for milk recording. In this method, the amount of milk is determined at least 11 times per year (the so-called test-days) from each cow during 24 hours and the total milk sample is collected, which is also used for milk composition analysis [17]. The data were collected between 2009 and 2017 from a total of 965 primiparous cows, which accounted for approximately 29% to 35% of the annual number of cows on the farm. Primiparae were assigned to age-season groups with two age categories (since the primiparous cows under local conditions have slightly different lactation curve shapes; those calving before 26 months of age have lower lactational yields than the animals beginning production at an older age [18]).

The following age groups were distinguished: younger primiparae, calving at the age of 20 to 26 months, older primiparae, calving at the age of 27 to 32 months. In addition, four calving seasons were defined: i) primiparae calving in January, February and March, ii) primiparae calving in April, May and June, iii) primiparae calving in July, August and September, iv) primiparae calving in October, November and December.

A total of eight age-season groups were created in this way (whose sample-size allowed for estimating model parameters and training). Lactation was divided into ten lactation stages of approximately 30 days each (except for the first lactation stage [from 5 to 30 days in milk] and the tenth lactation stage [from 270 to 305 days in milk]). Only the milk yields of more than 5 kg were included in the analysis. The milk yields recorded during the successive lactation stages were averaged over each age-season group. For instance, the average milk yields (kg/d) from 18 younger primiparae in their first lactation stage that were recorded in the first season of 2016 are presented in Table 1. These milk yields were averaged giving 28.49 kg of milk for the group of younger primiparae in the first calving season of 2016. The same method was applied to calculate the milk yields for subsequent lactation stages and obtain the values for the whole averaged lactation in the primiparae from a given age-season group.

The period of ten lactation stages was treated as one production cycle with a seasonal lag of 10, which did not always correspond to the production year of a primiparous cow. If the cow started production in the third or fourth season, her subsequent milk yields were recorded in the next year. However, to avoid confusion, the year-wise nomenclature was adopted by treating the years 2009 to 2010 as the year 2009, the years 2010 to 2011 as the year 2010 etc. (Supplementary Table S1). The numbers of younger primiparae in the analyzed years and seasons ranged from 10 to 18 and those of older primiparae ranged from 10 to 20 (Supplementary Table S1).

In the study period, the animals were housed in a free-stall barn under similar production conditions over the studied years and fed a standard diet, consisting of concentrate, green forage or silage. They also had access to outside runs. The average milk yield of the primiparae during the analyzed period amounted to approximately 9,000 kg over the standard lactation (Supplementary Table S2); however younger cows had lower yield. The records from the years 2009 to 2015 were used for the identification and parameter estimation of Wood’s and SARIMA models and for the development of the NARX artificial neural networks (training and validation data sets). The data from 2016 were utilized for the comparison of the real values with the predictions generated by the SARIMA and NARX models and those produced by Wood’s reference model (testing data set).

### The fitting and selection of the SARIMA models

The general multiplicative SARIMA model (*p*, *d*, *q*)×(*P*, *D*, *Q*) can be represented with the following expression [19]:

$$\varphi_{p}(B)\Phi_{P}(B^{S}){\left(1-B\right)}^{d}{\left(1-B^{S}\right)}^{D}Z_{t}=\theta_{q}(B)\Theta_{Q}(B^{S}){ɛ}_{t},$$

where, *φ_p_*(*B*) is the autoregressive operator = 1 − *φ*_1_*B* − *φ*_2_*B*^2^ − … − *φ_p_B^p^*, *θ_q_*(*B*) is the moving average operator = 1 − *θ*_1_*B* − *θ*_2_*B*^2^ − … − *θ_q_B^q^*, Φ*_P_*(*B^S^*) is the seasonal autoregressive operator = 1 − Φ*_S_B^S^* − Φ_2_*_S_B*^2^*^S^* − … − Φ*_PS_B^PS^*, Θ*_Q_*(*B^S^*) is the seasonal moving average operator = 1 − Θ*_S_B^S^* − Θ_2_*_S_B*^2^*^S^* − … − Θ*_QS_B^QS^*, *Z_t_* is the time series data consisting of the successive observations at time *t*, *ɛ_t_* is the residual error at time *t*, *B* is the backward shift operator, for which *B^k^Z_t_* = *Z_t_*_−_*_k_*, *S* is the seasonal length, *k* is the lag order, *p* is the non-seasonal autoregressive order, *d* is the order of non-seasonal differencing, *q* is the non-seasonal moving average order, *P* is the seasonal autoregressive order, *D* is the order of seasonal differencing, *Q* is the seasonal moving average order.

The input data series, comprising the mean milk yields at successive lactation stages from the years 2009 to 2015 was log-transformed in order to obtain variance homogeneity. The fitting of the SARIMA process model consisted of identification, parameter estimation and the verification of model goodness of fit. For the identification of the SARIMA models, i.e. the selection of the appropriate parameter values, the autocorrelation correlograms (the correlation of the data series with its lagged values, assuming the maximum value of 20) and partial autocorrelation correlograms (the partial correlation of the data series with its lagged values, after eliminating the effect of any correlations due to the terms at shorter lags – see correlograms in Supplementary Figures S1, S2) were used. In the correlogram analysis, the suggestions by Pankratz [20], Box and Jenkins [8], and Box et al [21] were taken into account. The processes of increasingly high *p* and *q* parameters were fitted until the moment, when the residuals from such a fitted process did not show any autocorrelation. The suggestion made by the above-mentioned authors about the adequacy of the models whose *p*+*q*<3 and *P*+*Q*<3 was considered.

The autocorrelation functions were also analyzed, paying attention to the statistical significance of the joint criterion of the so-called Ljung-Box Q statistics [22] for the maximum lag (20 in the present study), which indicates the model goodness of fit and follows approximately a chi-square distribution with *20*−*p*−*q* degrees of freedom (see correlograms in Supplementary Figures S1, S2). The lack of statistical significance indicates that the process is not a white noise. If the statistics was significant, such models (as unfitted) were excluded.

The above-mentioned statistics was estimated according to the following formula:

$$Q_{k}=n(n+2)\sum_{k=1}^{S}{\left(n-k\right)}^{-1}r_{k}^{2},$$

where *r^2^_k_* is the residual autocorrelation function:

$$r_{k}=\frac{\sum_{t=k+1}^{n}\left(x_{t}-\bar{x})(x_{t-k}-\bar{x}\right)}{\sum_{t=1}^{n}{\left(x_{t}-\bar{x}\right)}^{2}},$$

where, *S* is the maximum lag of this function, *x_t_* is the observation at time *t*, *x_t−k_* is the observation at time *t−k*, *χ̄* is the mean observation value, *k* is the lag order, and *n* is the number of observations.

The correlograms were some sort of clue about the future model form. Therefore, the statistical significance of the parameters was verified during the selection of the SARIMA models. The parameter estimation was performed using the exact Melard method [23], and in some cases (when it failed), with the approximate McLeod and Sales method [24]. An additional estimated element of some models was a constant, which in these cases represented the intercept.

At the next stage of the study, the model goodness of fit was verified by determining whether the residuals were the white noise process, i.e. whether the autocorrelation function (ACF) or partial autocorrelation function (PACF) values were statistically non-significant. The normal distribution of residuals was verified with the Shapiro-Wilk W test.

In the final selection of the SARIMA model for prediction, different error criteria were taken into account. The model with the lowest mean absolute percentage error (MAPE), mean absolute error (MAE) and root-mean-square error (RMSE) was selected. The following formulae were used for error calculation:

$$\begin{array}{l} RMSE=\sqrt{\frac{\sum_{t=1}^{n}{\left(Z_{t}-{\hat{Z}}_{t}\right)}^{2}}{n}},\\ MAPE=\frac{\sum_{t=1}^{n}\left|\frac{Z_{t}-{\hat{Z}}_{t}}{Z_{t}}\right|}{n}\cdot 100\%,\\ MAE=\frac{\sum_{t=1}^{n}\left|Z_{t}-{\hat{Z}}_{t}\right|}{n}, \end{array}$$

where, *Z_t_* is the observed value at time *t*, *Ẑ*_t_ is the predicted value at time *t*, *n* is the overall number of the observed values.

In addition, the Akaike information criterion (AIC) and its modified version (AIC_c_) [25] were considered in the selection of the best prediction model. The AIC and AIC_c_ values were calculated using the following formulae:

$$\begin{array}{l} AIC=2k+n\cdot \text{log}\left(\frac{RSS}{n}\right),\\ {AIC}_{c}=AIC+\frac{2k(k+1)}{n-k-1}, \end{array}$$

where *k* is the number of model parameters (*p*+*q*+*P*+*Q*+1) and *RSS* is the residual sum of squares:

$$RSS=\sum_{i=0}^{n}{\left(Z_{i}-{\hat{Z}}_{i}\right)}^{2},$$

where *Z_i_* is the real value, *Ẑ_i_* is the predicted value, *n* is the sample size (the number of observations).

### The construction and selection of the NARX artificial neural networks

In the selection of an appropriate NARX artificial neural network, the training and validation data sets (280 records in total) were used. A testing set included records from the years 2016 to 2017 (40 observations – the same ones that were utilized for the verification of the SARIMA and Wood’s models). Using a network automatic creator, two neural networks were constructed: the first one for younger primiparae and the second one for older primiparae, including an additional qualitative SEASON variable. This variable categorized data according to the criterion characteristic of the SARIMA models by distinguishing four calving seasons based on a cow’s calving date (the first, second, third or fourth calving season), without the necessity of dividing the dataset into seasonal groups. In the neural network construction, a training set and a validation set of 60 records were created. The validation set was used for the current monitoring of the training process in order to prevent over-fitting. The networks for younger and older primiparae were trained with the Broyden-Fletcher-Goldfarb-Shanno variable-metric algorithm, which approximated the Hessian inverse in the successive iterations based on its values from the previous step. This is an optimization algorithm used for multidimensional problems. It is characterized by a high effectiveness in the approximation of the second-order partial derivative matrices and achieves convergence very quickly, although it may be computationally intensive (it is usually used for networks with fewer than a thousand weights). It is considered one of the best optimization techniques [26,27]. For each verified network, a given number of iterations were carried out until obtaining the lowest RMSE, MAPE, and MAE values on the validation set. In addition, the network automatic creator selected the appropriate numbers of neurons in the hidden layer and the types of activation functions in the hidden and output layers (linear, exponential, logistic, or hyperbolic tangent).

### The use of Wood’s model

Based on lactational milk yields from the years 2009 to 2015 (like for the previous models), the parameters of Wood’s models were estimated with the quasi-Newton method separately for younger and older primiparae and for the same four calving seasons. The gamma function proposed by Wood [28] was used for characterizing lactation course:

$$Z=a\cdot t^{b}\cdot e^{-c\cdot t},$$

where, *Z* is the average daily milk yield at a given lactation stage, *t* is the time expressed in days, *e* is the natural logarithm base, *a* is the scale parameter that regulates the general height of the curve, *b* is the parameter controlling the increasing part of the lactation curve, *c* is the parameter associated with the decreasing part of the curve.

The individual Wood’s models were subsequently used for generating the prediction for the year 2016 and this prediction was compared with the real values.

### The verification of the predictions generated by the SARIMA, NARX, and Wood’s models

The next stage of the present study was to compare the predictions for individual age groups and calving seasons generated by the best (according to the adopted criteria) SARIMA models with the real values from 2016 to 2017 and the analogous predictions for individual age groups and calving seasons generated by NARX with the real milk yields at successive lactation stages. The same procedure was applied to Wood’s models. The RMSE, MAPE, and MAE values were compared for the predictions generated by all models.

Also, the Pearson correlation coefficients between the values predicted by the SARIMA, NARX, and Wood’s models and the real values were estimated. In addition, the significance of the differences in the correlation coefficients between individual models was verified using the t-test according to the following formula:

$$t=\frac{\text{ln}\;\left(\frac{1+r_{1}}{1-r_{1}}\right)-\text{ln}\;\left(\frac{1+r_{2}}{1-r_{2}}\right)}{2\sqrt{\frac{1}{n_{1}-3}+\frac{1}{n_{2}-3}}},$$

where, *r_1_* and *r_2_* are the compared correlation coefficients, *n_1_* and *n_2_* are the corresponding sample sizes, *t* is the value of the Student’s t statistics (*α* = 0.05 and *n_1_* + *n_2_* − 4 degrees of freedom were adopted).

Moreover, the Theil *I^2^* coefficient was calculated according to the following equation [29]:

$$I^{2}=\frac{\sum_{i=1}^{n}{\left(Z_{i}-\hat{Z}\right)}^{2}}{\sum_{i=1}^{n}Z_{i}^{2}}.$$

It can be decomposed into the sum of three coefficients determining the different sources of errors:

$$I^{2}=I_{O}^{2}+I_{B}^{2}+I_{E}^{2},$$

where, *I^2^_O_* is the term representing the prediction bias:

$$I_{O}^{2}=\frac{{\left({\bar{Z}}_{i}-{\hat{Z}}_{m}\right)}^{2}}{\frac{1}{n}\sum_{i=1}^{n}Z_{i}^{2}},$$

where, *Z̄_i_* is the mean real value, and *Ẑ_m_* is the mean predicted value, *I^2^_B_* represents the error caused by inadequate prediction flexibility:

$$I_{B}^{2}=\frac{{\left(\sigma_{i}-\sigma_{p}\right)}^{2}}{\frac{1}{n}\sum_{i=1}^{n}Z_{i}^{2}},$$

where, *σ_i_* is the standard deviation of the series of real values and *σ_p_* is the standard deviation of the series of predicted values.

*I^2^_E_* represents the error resulting from the insufficient convergence between the directions of predicted changes and the changes in the predicted variable:

$$I_{E}^{2}=\frac{2\sigma_{i}\sigma_{p}(1-r)}{\frac{1}{n}\sum_{i=1}^{n}Z_{i}^{2}},$$

where the symbols are defined as above and *r* is the correlation coefficient between the milk yields predicted by a given model and the real values.

In order to check whether the predictions generated by the SARIMA, NARX, and Wood’s models differed between each other, the Diebold-Mariano (DM) test with the small sample correction proposed by Harvey, Leybourne, and Newbold (*HLN*) was used:

$$\begin{array}{l} HLN=DM\sqrt{\frac{n+1-2h+n(n-1)}{n}},\\ DM=\frac{\bar{d}}{\sqrt{\frac{\gamma_{0}+2\sum_{t=1}^{h-1}\gamma_{t}}{n}}},\\ h=\sqrt[{3}]{n}+1,\\ \bar{d}=\frac{1}{n}\sum d_{i},\\ \gamma_{t}=\frac{1}{n}\sum_{i=t+1}^{n}(d_{i}-\bar{d})(d_{i-t}-\bar{d}),\\ d_{i}={es}_{i}^{2}-{en}_{i}^{2}, \end{array}$$

where, *es* is the difference between the real values and those predicted by a given model, *en* is the difference between the real values and those predicted by the model being compared.

Estimation and training of all models as well as the calculation of performance measures (except for DM) were carried out using Statistica 13.3 (TIBCO Software Inc., Tulsa, OK, USA). DM was calculated using R (R Development Core Team, R Foundation for Statistical Computing, Vienna, Austria) software.

## RESULTS

### The SARIMA model selection

For the detailed analysis and prediction, one SARIMA model was selected from each season. Four SARIMA models characterized by the lowest errors are presented in Supplementary Table S3. From among them, the best model (according to the lowest AIC and AIC_c_ values) for each season was selected for the comparison with the remaining models (Table 2). All the parameters of the presented SARIMA models selected for prediction were statistically significant (Supplementary Table S4). The Ljung-Box Q-statistics was not significant for any of them. The ACF graph (Supplementary Figures S1A, S1B) for the SARIMA models selected using the correlograms shows the exponential decay of the autocorrelation coefficients. Practically, they did not exceed the assumed 95% confidence level for any lag. Since the values of the Ljung-Box Q-statistics for the individual models were statistically non-significant for the last lag (*k* = 20), it can be stated that this was not white noise. The PACF values did not exceed the assumed confidence level either. Only the correlograms were not so ideal in this case (Supplementary Figures S2A, S2B).

### The selection of the NARX networks for prediction

**Figure 1:**
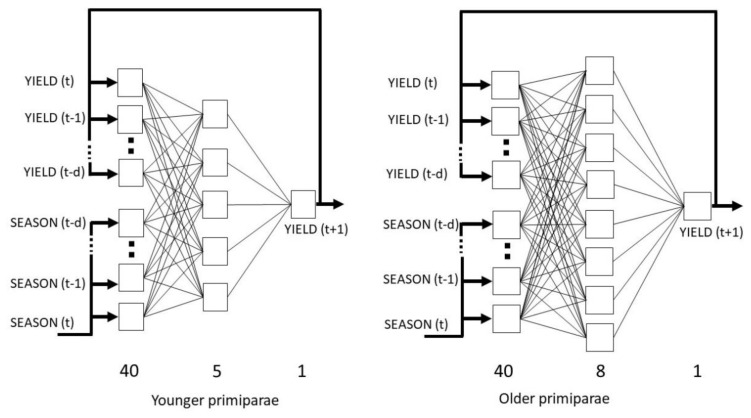
The architectures of the nonlinear autoregressive exogenous (NARX) artificial neural networks used for the prediction of milk yield at successive lactation stages in the younger and older cows, *t* is a time unit corresponding to lactation stage, *d* is the number of delays.

In order to determine the appropriate neural model for younger cows, the network with the lowest RMS error on the validation set (2.87 kg) was selected out of 40 analyzed networks. The exponential and linear activation functions were used in the hidden and output layers, respectively. The best network had a 40-5-1 architecture (the number of neurons in the input, hidden and output layers, respectively). A total of 48 iterations were carried out. For older cows, the error of the selected network on the validation set was 3.69 kg. The logistic activation functions were used both in the hidden and output layers. The network was trained for a total of 19 iterations and had a 40-8-1 architecture. The network architectures are presented in Figure 1.

### Wood’s model estimation

The parameters obtained for individual calving seasons in the groups of younger and older cows are presented in Table 3. All the estimates of the Wood’s model parameters were statistically significant (p<0.01) and greater than zero (Table 3).

### Comparison between the predictions generated by the investigated models

We did not find any statistically significant differences between the predictions generated by the investigated models (SARIMA, NARX, and Wood’s) and the real milk yields (the differences between the real milk yields and the predictions generated by the SARIMA and NARX models [in kg and %], the relative and absolute deviations calculated for lactation stages in individual cow age groups, calving seasons and lactation stages are presented in Supplementary Tables S5a, S5b, S6a and S6b, respectively).

The MAE values for younger cows in the first and second season were lowest for Wood’s models (0.9 kg and 1.29 kg, respectively), and slightly higher for the SARIMA (1.07 kg and 1.43 kg, respectively) and NARX (1.08 kg and 1.66 kg, respectively) models. An analogous situation occurred for the MAPE values, which amounted to 3.30% and 4.72% in the first and second season, respectively, for Wood’s models, 4.45% and 5.82%, respectively, for SARIMA as well as 4.14% and 6.51%, respectively, for NARX. The RMSE were slightly more diverse in these seasons (1.03 kg and 1.53 kg, respectively for Wood’s models, 1.37 kg and 1.56 kg, respectively for SARIMA as well as 1.24 kg and 1.98 kg, respectively for NARX; Table 4).

In the third and fourth season, we found the lowest MAE values for the SARIMA models (1.23 kg and 1.55 kg, respectively), slightly higher for Wood’s (1.49 kg and 2.43 kg, respectively) and the NARX (1.58 kg and 2.64 kg, respectively) models. An analogous situation occurred for the MAPE values (5.1% and 6.82% in the third and fourth season, respectively, for SARIMA, 5.44% and 9.24%, respectively for Wood’s model as well as 6.33% and 11.06%, respectively for NARX) and the RMSE values (1.54 kg and 1.86 kg, respectively for SARIMA, 1.77 kg and 2.63 kg, respectively for Wood’s model as well as 1.78 kg and 2.86 kg, respectively for NARX; Table 4). In older cows, the lowest MAE, MAPE, and RMSE values were characteristic of Wood’s models, only in the second season, SARIMA had slightly lower MAE values. We found the largest errors for the NARX networks (Table 4).

The low values of the Theil coefficients (Table 5; coefficients multiplied by 10^3^ for better legibility) confirmed a good prediction quality. In younger cows, we found the lowest Theil coefficients for Wood’s models in the first two seasons (1.27 and 2.86, respectively). The values for the SARIMA and NARX models in these two seasons were 2.24 and 2.99 as well as 1.84 and 4.80, respectively. In the third and fourth season, we observed the lowest coefficients for the SARIMA models (2.81 and 4.81, respectively), slightly higher for Wood’s model (3.72 and 9.59, respectively), and the highest ones for NARX (3.79 and 11.40, respectively). For all models, the greatest component of the Theil coefficient was *I^2^_E_* in the first and third season. In the second and fourth season, *I^2^_O_* played a larger role for the NARX and Wood’s models. We found the lowest contribution of the *I^2^_B_* coefficient for almost all models in younger cows (except for the SARIMA and Wood’s models in season 1).

In older cows (seasons 1 and 2), we noticed the lowest Theil coefficients for the SARIMA models (3.51 and 0.43, respectively), whereas higher ones for the Wood’s (5.12 and 0.93, respectively) and NARX (7.60 and 1.50, respectively) models. In the third season, this coefficient was slightly higher (21.73 for SARIMA, 16.23 for Wood’s, and 17.81 for NARX). In the fourth season, we found the lowest coefficient for SARIMA (6.11), and slightly higher for NARX (7.38) and Wood’s model (10.43). For the investigated models, *I^2^_E_* had the greatest contribution to the Theil coefficient. Only in the first season, *I^2^_0_* played a larger role for the NARX and Wood’s models. We found a greater contribution of the *I^2^_B_* coefficient (compared with *I^2^_0_*) in the second and fourth season (for all models) and the fourth season (for SARIMA).

**Figure 2:**
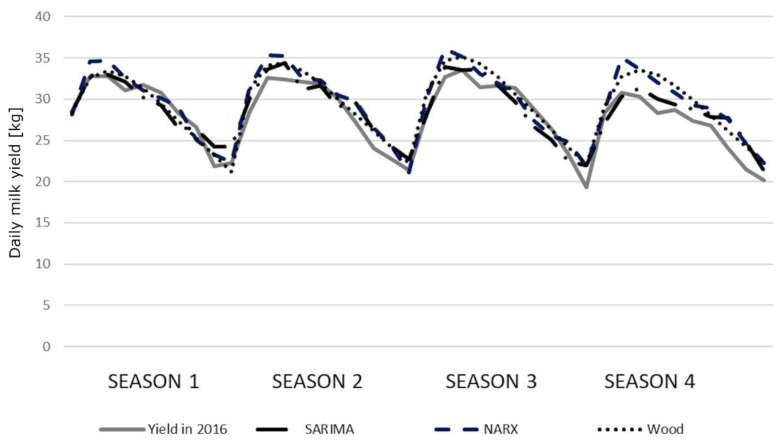
The course of the prediction generated by the seasonal auto-regressive integrated moving average (SARIMA) model, the nonlinear autoregressive exogenous (NARX) artificial neural networks and Wood’s models for younger cows relative to the real average milk yields in 2016.

**Figure 3:**
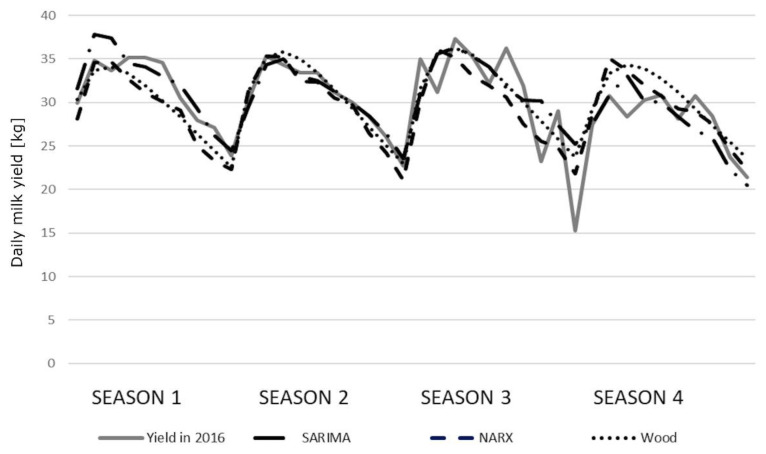
The course of the prediction generated by the seasonal auto-regressive integrated moving average (SARIMA) model, the nonlinear autoregressive exogenous (NARX) artificial neural networks and Wood’s models for older cows relative to the real average milk yields in 2016.

The correlation coefficients between the predicted and real milk yields were high and statistically significant (Table 6). Only the correlations for older cows (season 3) were lower (0.7500, 0.7827, and 0.8081 for the SARIMA, NARX, and Wood’s model, respectively). In general, we observed little higher correlation coefficients for Wood’s models. All the coefficients were statistically significant, although no significant differences were found among the models. The course of the prediction generated by individual models in younger cows was quite similar to the course of the real milk yield. Only in the fourth season, the values were overestimated by the NARX and Wood’s models (Figure 2). We observed slightly larger deviations in older cows, especially in the third and fourth season (Figure 3). The HLN test did not show any statistically significant differences in the predictions generated by different models.

## DISCUSSION

The application of the SARIMA models or the NARX artificial neural networks may be an interesting alternative to other methods of average milk yield estimation at successive lactation stages, such as Wood’s model [11,30]. In the light of our study and the available literature [14–16,31], the usefulness of different statistical methods for solving problems related to milk yield prediction could be confirmed. The fitting of the SARIMA models required the determination of the *p*, *d*, *q*, and *P*, *D*, *Q* parameters based on the autocorrelation and PACFs, parameter estimation and the goodness-of-fit verification. The correlogram analysis itself is not an ideal tool for the accurate model parameter selection, since the correlograms do not always unequivocally indicate the adequate choice of the *p*, *q*, and *P*, *Q* parameters. In the selection of the SARIMA model for specific prediction, AIC and AIC_c_ can be used. It is worth noting that, in most cases, the models with the low AIC values were also characterized by the low MAE, RMSE, and MAPE values. As stated by Box et al [21], the fitting of the processes with the increasing values of the *p* and *q* or *P* and *Q* parameters until the moment, when the residuals of such a fitted process do not show autocorrelation, can be limited to the value of *p*+*q*/(*P*+*Q*)<3. The investigated SARIMA models did not differ considerably in the number of parameters and the AIC and AIC_c_ values were mainly associated with the above-mentioned errors. It seems that the ultimate selection of the best predicting SARIMA model can be based on RMSE, MAE, and MAPE. The AIC measure, including the number of model parameters, is not more accurate, since the models usually did not differ between each other by more than one or two parameters. In the case of artificial neural networks, the number of parameters was incomparably higher than that for the SARIMA models. Therefore, the comparison between SARIMA and NARX using AIC and AIC_c_ was not performed, similarly as for Wood’s model.

It can be stated that the predictions presented in our study reflected the course of real milk yields during the distinguished lactation stages quite well. Adopting the 30-day lactation stages, some deviations expressed in kilograms ranged from 0.37 kg to 210 kg. The average deviations for the whole lactation ranged between 22.5 kg and 802 kg of milk. The performed analysis showed that the average predictions at successive lactation stages with the lowest deviations from the real milk yields in younger cows were generated by the SARIMA models, especially during the first lactation periods. At later stages, Wood’s and NARX models were more accurate with an increasing prediction horizon. In the case of older primiparae, this relationship became apparent in season 4, but it was not so evident for the remaining seasons. Wood’s models were more accurate, especially in a quite problematic season 3, similarly as the NARX models. This observation was further confirmed by the low values of the Theil coefficients for five SARIMA models and three Wood’s models. The NARX networks had slightly higher Theil coefficients. In the case of SARIMA (four models), NARX (five models) and Wood’s models (five models), the value of the Theil coefficient was mainly affected by the error (*I^2^_E_*) resulting from the insufficient convergence between the directions of prediction changes and the changes in the predicted variable. This indicates an insufficient prediction of turning points, which may lead to the prediction overestimation in some lactation periods (seasons 2 and 4 for younger cows and season 4 for older cows) and underestimation in others (season 1 and 3 for older cows) (Figures 2, 3). This finding corresponds to some extent with the results obtained by Murphy et al [15], who investigated lactational milk yield prediction at different time horizons and observed greater MAE and RMSE values for the NARX networks in the first prediction horizon (within 30 days in milk) but these errors decreased in the subsequent prediction periods. On the other hand, the static artificial neural networks were characterized by a general increase in the prediction accuracy with decreasing horizon (a reduction in RMSE from 12.0% to 10.7% with the prediction horizon changing from 305 to 10 days), although in the same prediction horizon range, the underestimation of lactation peak yield increased from 4.8% to 7.4%. However, when analyzing these predictions at 10-day intervals, the errors for NARX and the rest of the models were characterized by larger fluctuations – the neural networks had lower errors in some periods, and the remaining models in others. The predictions generated by the SARIMA, NARX, and Wood’s models in our study were more flexible, which was confirmed by the lowest contribution of the *I^2^_B_* term to the overall Theil coefficient. Moreover, a very low contribution of the *I^2^_O_* term (especially for the SARIMA models) indicated a low prediction bias. However, this coefficient predominated among the components of the Theil coefficient for three NARX and three Wood’s models, confirming a higher prediction bias for them.

A different trend in the error values was found in the prediction of monthly total dissolved solids in the Rio Grande [32], where the authors observed slightly lower RMSE and MAE values for the neural networks compared with the ARIMA model at the one-month prediction horizon, whereas this situation changed in the second and third month, when smaller errors were made by the ARIMA model. A similar relationship was found by Zhang et al [31], who recorded an increase in RMSE for the NARX model with lengthening prediction horizon. However, it should be remembered that the results from these studies are not directly comparable with ours and only some general trends can be observed.

The correlation coefficients between the predicted and real milk yields in our study were slightly higher than those obtained by Abudu et al [32], who recorded the values ranging from 0.78 to 0.89; however, these correlations were almost always greater for the neural networks compared with ARIMA, similarly as in our study. In the work by Zhang et al [31] on the use of an automatic system of model configuration and optimization for milk production prediction, the correlation coefficients exceeded 0.84 for all the nine developed models (multinomial, adaptive multinomial, Legendre’s multinomial, cubic splines, log-squared, multiple linear regression, static artificial neural networks, surface fitting and NARX), which confirmed their good overall quality. At a longer prediction horizon (amounting to 365 days), the correlation coefficients for these models ranged from 0.85 to 0.98 (r = 0.98 for the best surface fitting model and r = 0.85 for the worst multiple linear regression). The accuracy of the NARX model increased with a decreasing prediction horizon (the correlation rose from 0.96 to 0.98 with the horizon changing from 365 to 10 days). Also, in the study by Murphy et al [15] on the use of the NARX and static artificial neural networks, among others, for milk production prediction, the correlation coefficients generally slightly increased with a decreasing prediction horizon (from 305 to 10 days) and they were slightly higher for NARX compared with the static artificial neural networks each time (0.97 vs 0.94 to 305 days, 0.97 vs 0.94 to 50 days, 0.98 vs 0.95 to 30 days, and 0.98 vs 0.95 to 10 days for the NARX model and the static artificial neural networks, respectively). However, such a trend was not observed for the last investigated model, i.e. multiple linear regression, for which the values of the correlation coefficient were almost invariant.

We obtained exceptionally worse predictions (compared with other seasons) in the third season for older cows, which is also depicted in Figure 3. This most probably resulted from the large variations in the real milk yields of cows calving during this season. It is difficult to explicitly determine the source of these fluctuations but, in general, a sudden breakdown caused by e.g. diseases, the decreased nutritive value of feeds, staff changes and other unpredictable factors, may (although not necessarily) result in milk yield changes and, consequently, negatively affect prediction accuracy. It can also be noticed that the lactation peak was slightly earlier in season 1 (especially for younger cows) with a more rapid decrease in milk yield, which may indicate inappropriate feeding. In the remaining seasons, a second (although not so evident) lactation peak could be observed, which in turn may reflect additional feeding (in this case, supplementation of the diet with green forage). Considerable milk yield variation occurred in season 4, which again may indicate inappropriate feeding. The predictions do not reflect perturbations visible in the course of real milk yield (especially for SARIMA), which allows us to suppose that they resulted from some unpredictable factors occurring on the farm.

The predictions produced by the SARIMA models, rather poorly reflect seasonal fluctuations in the analyzed series and the predictions are increasingly closer to the trend line with a lengthening prediction horizon. It is probably associated with the fact that seasonality is only modelled by the stationary component of the model (no seasonal differencing). The SARIMA models poorly detect non-linearity in time series data, whereas artificial neural networks are more accurate in this regard, similarly as Wood’s model. The use of artificial neural networks for time series prediction, including additional factors such as calving season, makes it possible to quickly develop a prediction model. The fitting and selection of the best SARIMA model seems to be more time-consuming and absorbing. It requires the verification of many elements (correlograms, parameter significance, the normal distribution of residuals etc.). There are no such requirements for the neural networks, and the use of the automatic network construction module greatly facilitates the development process. When fitting the SARIMA models, it was necessary (for higher accuracy) to divide the dataset into two age groups and four calving seasons, which gave a total of eight models for selection. If the dataset had been larger, it would have been possible to distinguish e.g. 13 age groups (20 to 32 months) instead of just two, which would have resulted in a more accurate prediction, but also the number of the SARIMA models to select from would have increased to 52. The selection itself caused the necessity of verifying many combinations of the *p*, *q*, and *P*, *Q* parameters. The advantage of the NARX networks in this regard is evident. Finally, the development of Wood’s models was easier compared with other models (NARX and SARIMA) presented in our study.

In conclusion, the applied SARIMA and NARX models predicted the average milk yields at successive stages of the standard lactation of primiparous cows quite well, similarly as the frequently used Wood’s model. It is difficult to indicate the most accurate model in our study, since the predictions did not differ significantly from the real values and among themselves. It is only possible to indicate a slight superiority of individual models. The SARIMA models slightly better predicted the values in the initial prediction periods, whereas NARX and Wood’s models produced more accurate predictions in the later periods, but the indication of the more accurate model is doubtful since the predictions did not differ significantly from the observed values or among each other. The application of the SARIMA model was more time-consuming than that of NARX and Wood’s model.

The application of the prediction methods presented in our study may, to some extent, allow farmers to estimate the milk yield of cows that begin milk production for the first time, providing the basis for the rational management of farm resources, a better definition of tasks and a clearer presentation of the reasons behind each decision made. However, the implementation of such models in user-friendly farm management software would be required first.

## Figures and Tables

**Table 1:** Average milk yields (kg/d) from 18 younger primiparae in their first lactation stage

Items	n	Daily milk yield (kg)							Total	Mean
January	7	25.7	28.1	24.9	26.7	27.1	35.6	36.7	204.8	
February	6	32.8	32.5	26.6	29.2	31.4	21.9	-	174.4	
March	5	32.3	25.7	29.3	25.4	20.9	-	-	133.6	
Total	18								512.8	28.49

**Table 2:** The seasonal auto-regressive integrated moving average (SARIMA) models selected for further prediction according to the analyzed error, Q and AIC values

Season	Younger				Older			
	
	1	2	3	4	1	2	3	4
Model	(010)(200)	(100)(101)	(100)(101)	(110)(100)	(010)(200)	(010)(101)	(100)(200)	(010)(200)
MAE	1.06	1.43	1.23	1.55	1.47	0.53	2.60	1.48
RMSE	1.38	1.56	1.54	1.86	1.60	0.64	3.38	2.20
MAPE	3.98	5.03	4.37	5.73	4.54	1.77	8.23	5.39
Q	23.67	14.01	8.81	17.34	20.65	17.57	25.96	22.48
AIC	12.27	16.91	16.58	22.17	18.39	−4.97	32.34	21.57
AICc	16.27	24.91	24.58	26.17	22.39	−0.97	40.34	25.57

Q, Q statistics; AIC, Akaike information criterion; AIC_c_, corrected Akaike information criterion; MAE, mean absolute error; RMSE, root-mean-square error; MAPE, mean absolute percentage error.

**Table 3:** Estimates of the Wood’s model parameters and their standard errors

Items	Season 1			Season 2			Season 3			Season 4		
			
	*a*	*b*	*c*	*a*	*b*	*c*	*a*	*b*	*c*	*a*	*b*	*c*
Younger primiparare												
Est	32.44	0.39	0.13	34.84	0.29	0.11	35.08	0.35	0.13	32.68	0.34	0.12
SE	0.46	0.03	0.01	0.69	0.04	0.01	0.78	0.04	0.01	0.82	0.05	0.01
Older primiparare												
Est	33.92	0.31	0.11	35.36	0.35	0.12	35.59	0.34	0.12	32.74	0.36	0.12
SE	0.98	0.06	0.01	0.54	0.03	0.01	0.94	0.05	0.01	0.81	0.05	0.01

Est, estimate; SE, standard error.

**Table 4:** Errors for the selected SARIMA, NARX, and Wood’s models according to calving season and age group

Errors	Season 1			Season 2			Season 3			Season 4		
			
	SARIMA	NARX	Wood	SARIMA	NARX	Wood	SARIMA	NARX	Wood	SARIMA	NARX	Wood
Younger primiparae												
MAE (kg)	1.07	1.08	0.90	1.43	1.66	1.29	1.23	1.58	1.49	1.55	2.64	2.43
RMSE (kg)	1.37	1.24	1.03	1.56	1.98	1.53	1.54	1.78	1.77	1.86	2.86	2.63
MAPE (%)	4.45	4.14	3.30	5.82	6.51	4.72	5.10	6.33	5.44	6.82	11.06	9.24
Older primiparae												
MAE (kg)	1.60	2.39	0.92	0.54	1.03	0.56	2.60	3.82	1.70	1.48	1.83	0.88
RMSE (kg)	1.87	2.75	1.04	0.64	1.19	0.60	3.38	3.99	2.07	2.20	2.42	1.02
MAPE (%)	5.51	8.51	2.96	1.94	3.95	1.84	9.49	13.23	5.36	5.83	7.08	3.07

SARIMA, seasonal auto-regressive integrated moving average; NARX, nonlinear autoregressive exogenous artificial neural networks; MAE, mean absolute error; RMSE, root-mean-square error; MAPE, mean absolute percentage error.

**Table 5:** The Theil coefficients for the predictions generated by the SARIMA, NARX, and Wood’s models according to calving season and age group

Items	SARIMA	NARX	Wood	SARIMA	NARX	Wood
	**Younger primiparae**					
	**Season 1**			**Season 2**		
*I*^2^	2.23930	1.84090	1.27365	2.98790	4.80256	2.85991
*I*^2^_O_	0.00522	0.23076	0.02772	1.07631	2.94985	2.01053
*I*^2^_B_	0.62093	0.13207	0.03447	0.22519	0.15012	0.00052
*I*^2^_E_	1.61315	1.47820	1.21241	1.68686	1.70325	0.85028
*I*^2^_O_ (%)	0.23	12.54	2.18	36.02	61.42	70.30
*I*^2^_B_ (%)	27.73	7.17	2.71	7.54	3.13	0.02
*I*^2^_E_ (%)	72.04	80.30	95.19	56.46	35.47	29.73
	**Season 3**			**Season 4**		
*I*^2^	2.80554	3.78646	3.72267	4.81020	11.39751	9.59155
*I*^2^_O_	0.00001	1.14645	1.65111	2.07714	9.24165	8.12290
*I*^2^_B_	0.00002	0.01287	0.00167	0.56688	0.40283	0.13231
*I*^2^_E_	2.80548	2.62715	2.07094	2.16624	1.75400	1.33615
*I*^2^_O_ (%)	0.00	30.28	44.35	43.18	81.08	84.69
*I*^2^_B_ (%)	0.00	0.34	0.04	11.78	3.53	1.38
*I*^2^_E_ (%)	100.00	69.38	55.63	45.03	15.39	13.93
	**Older primiparae**					
	**Season 1**			**Season 2**		
*I*^2^	3.50576	7.59612	5.11710	0.42829	1.49970	0.92896
*I*^2^_O_	0.55068	4.52857	2.96390	0.01092	0.42183	0.03022
*I*^2^_B_	0.13301	0.17046	0.00000	0.11395	0.48113	0.27873
*I*^2^_E_	2.82201	2.89659	2.15318	0.30339	0.59728	0.62001
*I*^2^_O_ (%)	15.71	59.62	57.92	2.55	28.13	3.25
*I*^2^_B_ (%)	3.79	2.24	0.00	26.60	32.08	30.00
*I*^2^_E_ (%)	80.50	38.13	42.08	70.84	39.83	66.74
	**Season 3**			**Season 4**		
*I*^2^	21.73444	17.81869	16.23342	6.10826	7.37974	10.43485
*I*^2^_O_	1.10261	1.03546	0.32794	0.20497	1.62102	4.75037
*I*^2^_B_	7.99121	3.79733	5.18000	0.62420	0.63255	0.37121
*I*^2^_E_	12.64039	12.98628	10.72585	5.28000	5.13140	5.31356
*I*^2^_O_ (%)	5.07	5.81	2.02	3.36	21.97	45.52
*I*^2^_B_ (%)	36.77	21.31	31.91	10.22	8.57	3.56
*I*^2^_E_ (%)	58.16	72.88	66.07	86.44	69.53	50.92

SARIMA, seasonal auto-regressive integrated moving average; NARX, nonlinear autoregressive exogenous artificial neural networks.

**Table 6:** The Pearson correlation coefficients between the real and predicted milk yields

Season	Younger primiparae			Older primiparae		
	
	SARIMA	NARX	Wood	SARIMA	NARX	Wood
1	0.9418	0.9621	0.9672	0.9172	0.9174	0.9303
2	0.9555	0.9643	0.9791	0.9892	0.9823	0.9807
3	0.9365	0.9420	0.9582	0.7500	0.7827	0.8081
4	0.9163	0.9548	0.9625	0.8116	0.8298	0.8156

SARIMA, seasonal auto-regressive integrated moving average; NARX, nonlinear autoregressive exogenous artificial neural networks.
