# Supplementary material for: Lactation milk yield prediction in primiparous cows on a farm using the seasonal auto-regressive integrated moving average model, nonlinear autoregressive exogenous artificial neural networks and Wood’s model
Source: Anim Biosci. 2020 Apr 12;34(4):770–82. doi: 10.5713/ajas.19.0939 (PMC7961269; doi:10.5713/ajas.19.0939)
Supplement: Supplementary file 1 [file ajas-19-0939-suppl.pdf]

Supplementary Table S1. Primiparae from individual age-season groups during the investigated lactation periods

Year[1]	Year U[2]	Age	Season 1	Season 2	Season 3	Season 4	Σ
2009	2009-2010	Younger	12	16	17	17	62
		Older	10	18	18	16	62
2010	2010-2011	Younger	17	12	15	15	59
		Older	16	15	13	12	56
2011	2011-2012	Younger	18	13	16	16	63
		Older	20	17	19	17	73
2012	2012-2013	Younger	18	15	17	14	64
		Older	17	11	18	13	59
2013	2013-2014	Younger	16	15	18	18	67
		Older	14	14	13	16	57
2014	2014-2015	Younger	14	13	15	15	57
		Older	19	18	14	13	64
2015	2014-2015	Younger	15	13	10	11	49
		Older	13	11	12	11	47
2016	2016-2017	Younger	18	16	16	12	62
		Older	16	17	13	18	64
		Σ	253	234	244	234	965

[1]Year – the year adopted for lactation duration during the investigated period.

[2]Year U – the real time of test-day recording.

Supplementary Table S2. Average 305-day milk yields of first-lactation cows according to age group and calving season between 2009 and 2015

Season	Younger		Older	
	Mean	SD	Mean	SD
1	8723	1031	8955	1052
2	9047	1081	9288	1072
3	9220	1123	9489	1111
4	8757	1117	9076	1184
Total	8926	1085	9192	1101

Supplementary Table S3. The seasonal auto-regressive integrated moving average (SARIMA) models, from which the best ones were selected for prediction (the best model for each season is underlined)

	MAE[1]	*RMSE*[2]	*MAPE*[3]	*Q*[4]	*AIC*[5]	AIC_c[6]
			Younger Primiparae			
			Season 1			
(010) (200)	1.06	1.38	3.98	23.67	12.27	16.27
(010) (100)	1.11	1.52	4.10	22.96	14.04	16.35
(011) (200)	1.15	1.33	4.16	20.22	13.70	21.70
(100) (100)	1.16	1.36	4.37	15.67	13.25	17.25
			Season 2			
(010) (200)	1.92	2.37	7.20	19.72	23.28	27.28
(100) (100)	1.49	1.76	5.42	27.17	21.46	25.46
(100) (101)	1.43	1.56	5.03	14.01	16,91	24.91
(100) (200)	1.49	1.76	5.19	15.52	17.37	25.37
			Season 3			
(011) (100)	4.51	4.90	19.22	14.04	37.20	41.20
(100) (001)	2.19	2.71	8.05	34.85	25.92	29.92
(100) (100)	2.15	2.37	8.46	13.18	23.45	27.45
(100) (101)	1.23	1.54	4.37	8.81	16.58	24.58
			Season 4			
(100) (002)	4.13	4.49	13.60	19.53	36.60	44.60
(100) (100)	2.86	3.17	9.91	17.40	29.11	33.11
(100) (101)	3.16	3.39	10.80	20.99	32.45	40.45
(110) (100)	1.55	1.86	5.73	17.34	22.17	26.17

Supplementary Table S3. Cont.

	MAE	*RMSE*	*MAPE*	*Q*	*AIC*	AIC_c
			Older Primiparae			
			Season 1			
(001) (200)	1.44	1.70	4.55	29.96	18.62	26.62
(010) (200)	1.47	1.60	4.54	20.65	18.39	22.39
(100) (101)	3.01	3.38	10.51	33.65	32.36	40.36
(100) (102)	2.03	2.46	6.94	18.12	28.06	43.06
			Season 2			
(001) (100)	1.50	1.72	5.07	19.60	18.22	22.22
(010) (100)	3.34	3.95	12.29	16.63	30.19	31.10
(010) (101)	0.53	0.64	1.77	17.57	-4.97	-0.97
(100) (101)	0.93	1.03	3.15	18.35	19.41	27.41
			Season 3			
(001) (002)	4.06	4.74	13.87	24.32	37.16	45.16
(100) (101)	2.79	3.41	9.06	19.59	32.47	40.47
(100) (200)	2.60	3.38	8.23	25.96	32.34	40.34
(111) (101)	2.36	3.22	7.34	15.45	33.40	48.40
			Season 4			
(010) (002)	2.78	3.02	10.85	21.08	28.10	32.10
(010) (200)	1.48	2.20	5.39	22.48	21.57	25.57
(100) (002)	4.38	6.28	11.88	20.63	44.75	52.57
(100) (100)	2.72	3.64	8.20	12.71	31.78	35.78

[1]Mean absolute error.

[2]Root-mean-square error.

[3]Mean absolute percentage error.

[4]Q statistics.

[5]Akaike Information Criterion.

[6]Corrected Akaike Information Criterion.

Supplementary Table S4. The parameters of the seasonal auto-regressive integrated moving average (SARIMA) models selected for prediction

	Season		Constant	p(1)	q(1)	Ps(1)	Qs(1)	Ps(2)
Younger primiparae	1	(0,1,0) (2,0,0)				0.5116**	0.3211**	
	2	(1,0,0) (1,0,1)	3.3689**	0.4725**		0.9865**	0.7312**	
	3	(1,0,0) (1,0,1)	3.1176**	0.7140**		0.9526**	0.3398*	
	4	(1,1,0) (1,0,0)		-0.2594*		0.7974**		
Older primiparae	1	(0,1,0) (2,0,0)				0.2862*		0.6534**
	2	(0,1,0) (1,0,1)				0.9856**	0.6050**	
	3	(1,0,0) (2,0,0)	3.3921**	0.5171**		0.4074**	0.4885**	
	4	(0,1,0) (2,0,0)				0.5699**		0.3058*

* $p<0.05$ and **$p<0.01$

Supplementary Table S5a. The differences between the real milk yields and those predicted by the SARIMA, NARX and Wood's models (in kg and %) as well as the summary of the relative and absolute deviations calculated for each lactation stage according to calving season and lactation stage (younger cows)

Stage	Season 1							Season 2						
	Real	SARIMA[1]		NARX[2]		Wood		Real	SARIMA		NARX		Wood	
		kg	%	kg	%	kg	%		kg	%	kg	%	kg	%
1	28.49	0.05	0.16	0.36	1.25	0.06	0.22	28.41	-1.62	5.72	-2.9	10.21	-2.74	-9.64
2	32.82	-0.03	-0.1	-1.77	5.38	0.23	0.69	32.57	-1.06	3.27	-2.75	8.43	-1.55	-4.75
3	32.74	-0.23	-0.7	-1.96	5.98	-0.69	-2.12	32.44	-1.95	6.01	-2.76	8.51	-1.91	-5.87
4	31.09	-0.99	-3.18	-1.54	4.95	-1.67	-5.36	32.12	0.97	3.03	-0.33	1.04	-1.29	-4.00
5	31.77	1.6	5.03	0.66	2.07	0.46	1.44	31.85	0.24	0.75	-0.47	1.49	-0.03	-0.09
6	30.78	1.49	4.85	0.71	2.29	1.33	4.32	30.09	1.06	3.51	-0.46	1.52	0.03	0.09
7	28.28	1.75	6.17	-0.79	2.81	0.87	3.09	27.16	-2.42	8.92	-2.59	9.54	-0.95	-3.51
8	26.57	0.14	0.51	1.51	5.68	1.28	4.82	24.06	-2.2	9.13	-2.42	10.05	-2.07	-8.61
9	21.83	-2.37	-10.9	-1.45	6.66	-1.38	-6.31	22.69	-1.53	6.72	-1.55	6.84	-1.49	-6.58
10	22.22	-2.05	-9.24	-0.11	0.5	1.03	4.64	21.42	-1.29	6.01	0.36	1.66	-0.86	-4.04
Mean	28.66	1.05	4.08	1.08	3.76	0.90	3.30	28.28	1.43	5.31	1.66	5.93	1.29	4.72
∑RD[3]		-30.31		-134.1		50.5			-292.39		-459.87		-376.35	

∑AD[4]	330.88	324.26	225.2	428.63	484.77	377.99

[1]Seasonal auto-regressive integrated moving average.

[2]Nonlinear autoregressive exogenous artificial neural networks.

[3]The sum of the relative deviations for the whole lactation (kg).

[4]The sum of the absolute deviations for the whole lactation (kg).

Supplementary Table S5b. The differences between the real milk yields and those predicted by the SARIMA, NARX and Wood's models (in kg and %) as well as the summary of the relative and absolute deviations calculated for each lactation stage according to calving season and lactation stage (younger cows)

Stage	Season 3							Season 4						
	Real	SARIMA[1]		NARX[2]		Wood		Real	SARIMA		NARX		Wood	
		kg	%	kg	%	kg	%		kg	%	kg	%	kg	%
1	28.36	0.1	0.34	-1.82	6.41	-2.53	-8.93	28.29	1.19	4.2	-0.34	1.2	-0.78	-2.75
2	32.71	-1.17	3.58	-3.35	10.23	-1.97	-6.01	30.75	0.45	1.46	-4.32	14.06	-2.05	-6.67
3	33.53	0.02	0.05	-1.59	4.74	-1.66	-4.96	30.36	-0.96	3.16	-3.29	10.83	-3.18	-10.47
4	31.43	-2.2	7.01	-1.63	5.18	-2.84	-9.04	28.32	-1.71	6.05	-3.66	12.92	-4.61	-16.26
5	31.59	-0.07	0.21	-0.3	0.96	-1.04	-3.31	28.71	-0.64	2.22	-2.08	7.26	-2.90	-10.11
6	31.32	1.67	5.34	0.76	2.42	0.68	2.19	27.39	-1.43	5.22	-1.86	6.8	-2.55	-9.31
7	28.84	2.16	7.48	1.34	4.65	0.36	1.26	26.81	-1.01	3.78	-2.1	7.85	-1.27	-4.72
8	26.44	1.36	5.13	0.86	3.25	0.15	0.57	24.05	-3.67	15.27	-3.52	14.63	-2.09	-8.71
9	23.25	0.81	3.5	-1.55	6.67	-0.88	-3.76	21.47	-3.29	15.34	-3.19	14.87	-2.74	-12.78
10	19.27	-2.72	14.11	-2.56	13.29	-2.78	-14.41	20.19	-1.14	5.66	-2.07	10.24	-2.14	-10.60
Mean	28.67	1.23	4.67	1.58	5.78	1.49	5.44	26.63	1.55	6.24	2.64	10.07	2.43	9.24
∑RD[3]		-15.36		-298.84		-376.19			-378.41		-801.76		-736.07	

∑AD[4]	381.19	476.43	385.1	464.78	801.76	736.07

[1]Seasonal auto-regressive integrated moving average.

[2]Nonlinear autoregressive exogenous artificial neural networks.

[3]The sum of the relative deviations for the whole lactation (kg).

[4]The sum of the absolute deviations for the whole lactation (kg).

Supplementary Table S6a. The differences between the real milk yields and those predicted by the SARIMA, NARX and Wood's models (in kg and %) as well as the summary of the relative and absolute deviations calculated for each lactation stage according to calving season and lactation stage (older cows)

Stage	Season 1							Season 2						
	Real	SARIMA[1]		NARX[2]		Wood		Real	SARIMA		NARX		Wood	
		kg	%	kg	%	kg	%		kg	%	kg	%	kg	%
1	29.93	-1.62	5.43	1.80	6.00	-1.05	-3.51	30.24	0.75	2.49	-1.07	3.54	-0.42	-1.36
2	34.80	-2.99	8.60	0.21	0.62	2.10	6.03	35.31	0.99	2.81	-0.01	0.02	0.89	1.95
3	33.64	-3.74	11.12	-1.06	3.14	-0.33	-0.98	34.29	-0.73	-2.12	-0.91	2.65	0.75	1.15
4	35.19	0.66	1.87	2.56	7.28	-1.12	-3.35	33.42	0.45	1.35	0.97	2.89	-0.49	-1.40
5	35.14	1.10	3.12	4.03	11.46	-1.05	-3.07	33.44	0.93	2.80	1.12	3.34	-0.42	-0.95
6	34.60	1.58	4.56	4.53	13.08	-0.22	-0.72	31.17	-0.12	-0.37	0.62	2.00	-0.54	-0.32
7	30.52	-1.52	4.99	1.45	4.73	0.81	2.86	29.99	0.06	0.19	0.24	0.80	0.50	-1.71
8	27.98	-1.30	4.65	2.92	10.44	0.81	3.08	28.37	-0.10	-0.36	1.89	6.67	0.24	0.88
9	27.14	0.90	3.31	3.86	14.21	0.60	2.44	26.03	-0.39	-1.50	1.79	6.87	0.92	3.68
10	23.87	-0.57	2.40	1.54	6.45	-1.08	-4.77	22.73	-0.84	-3.68	1.67	7.33	-0.41	-1.80
Mean	31.28	1.60	5.00	2.39	7.74	0.92	3.08	30.50	0.54	1.77	1.03	3.61	0.56	1.52
∑RD[3]		-220.43		653.55		-15.86			22.49		202.93		30.44	

∑AD[4]	474.25	716.96	234.39	161.29	311.33	167.70

[1]Seasonal auto-regressive integrated moving average.

[2]Nonlinear autoregressive exogenous artificial neural networks.

[3]The sum of the relative deviations for the whole lactation (kg).

[4]The sum of the absolute deviations for the whole lactation (kg).

Supplementary Table S6b. The differences between the real milk yields and those predicted by the SARIMA, NARX and Wood's models (in kg and %) as well as the summary of the relative and absolute deviations calculated for each lactation stage according to calving season and lactation stage (older cows)

Stage	Season 3							Season 4						
	Real	SARIMA[1]		NARX[2]		Wood		Real	SARIMA		NARX		Wood	
		kg	%	kg	%	kg	%		kg	%	kg	%	kg	%
1	34.96	3.92	11.22	4.78	13.68	1.29	3.69	27.42	-0.08	0.28	-1.21	4.41	-0.60	-2.20
2	31.21	-4.39	14.07	-4.85	15.53	-0.44	-1.41	30.75	-0.13	0.42	-4.32	14.06	1.21	3.92
3	37.33	1.05	2.80	2.21	5.92	1.13	3.03	28.32	-4.77	16.85	-5.33	18.81	0.45	1.60
4	35.34	0.01	0.03	2.28	6.46	-0.84	-2.37	30.28	0.06	0.21	-1.70	5.61	-1.13	-3.73
5	35.79	1.73	4.84	3.90	10.89	-1.53	-4.27	30.84	0.79	2.55	0.05	0.15	-0.61	-1.98
6	36.24	4.56	12.57	5.68	15.67	3.41	9.41	28.13	-0.22	0.78	-1.12	3.99	-0.31	-1.11
7	31.93	1.64	5.13	4.43	13.88	1.35	4.23	30.77	4.00	12.99	1.86	6.03	1.26	4.09
8	23.21	-7.01	30.18	-2.37	10.21	0.75	3.23	28.47	2.50	8.79	0.90	3.16	1.79	6.29
9	29.03	1.66	5.71	4.23	14.57	1.22	4.20	23.74	1.40	5.91	-0.92	3.88	-0.19	-0.80
10	25.27	0.06	0.26	3.44	13.61	-2.00	-7.91	21.37	0.90	4.20	-0.89	4.16	-0.50	-2.35

Mean	32.03	2.60	8.68	3.82	12.04	1.40	4.38	28.01	1.48	5.30	1.83	6.43	0.81	2.81
∑RD[3]		77.74		705.26		113,82			138.45		-379.01		41,28	
∑AD[4]		761.45		1138.28		320.15			449.52		547.19		218.03	

[1]Seasonal auto-regressive integrated moving average.

[2]Nonlinear autoregressive exogenous artificial neural networks.

[3]The sum of the relative deviations for the whole lactation (kg).

[4]The sum of the absolute deviations for the whole lactation (kg).

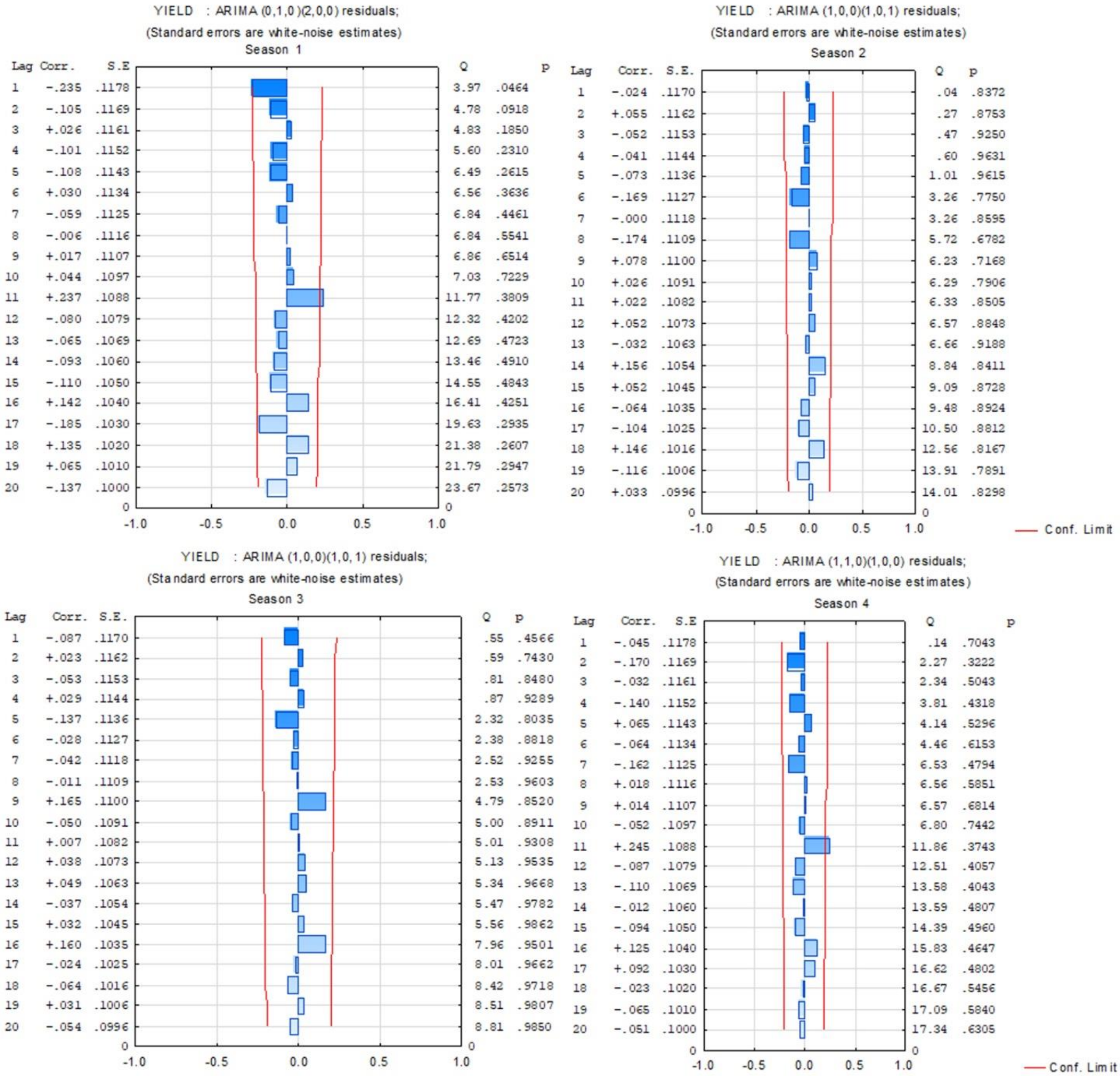

Supplementary Fig. S1A. The correlograms of the autocorrelation functions for the best selected SARIMA models for the younger primiparae (at the age of 20 – 26 months)

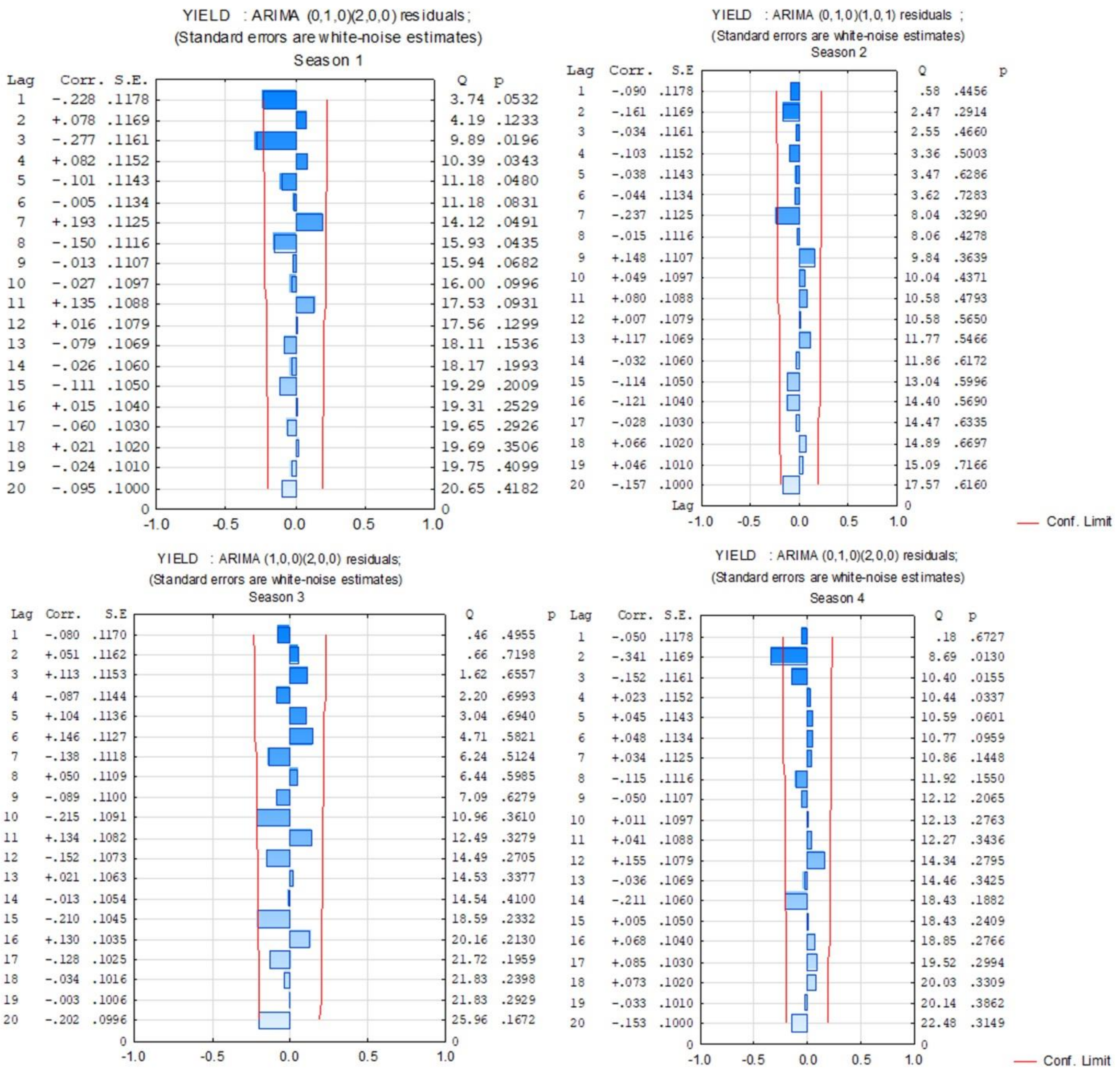

Supplementary Fig. S1B. The correlograms of the autocorrelation functions for the best selected SARIMA models for the older primiparae (at the age of 27 – 32 months)

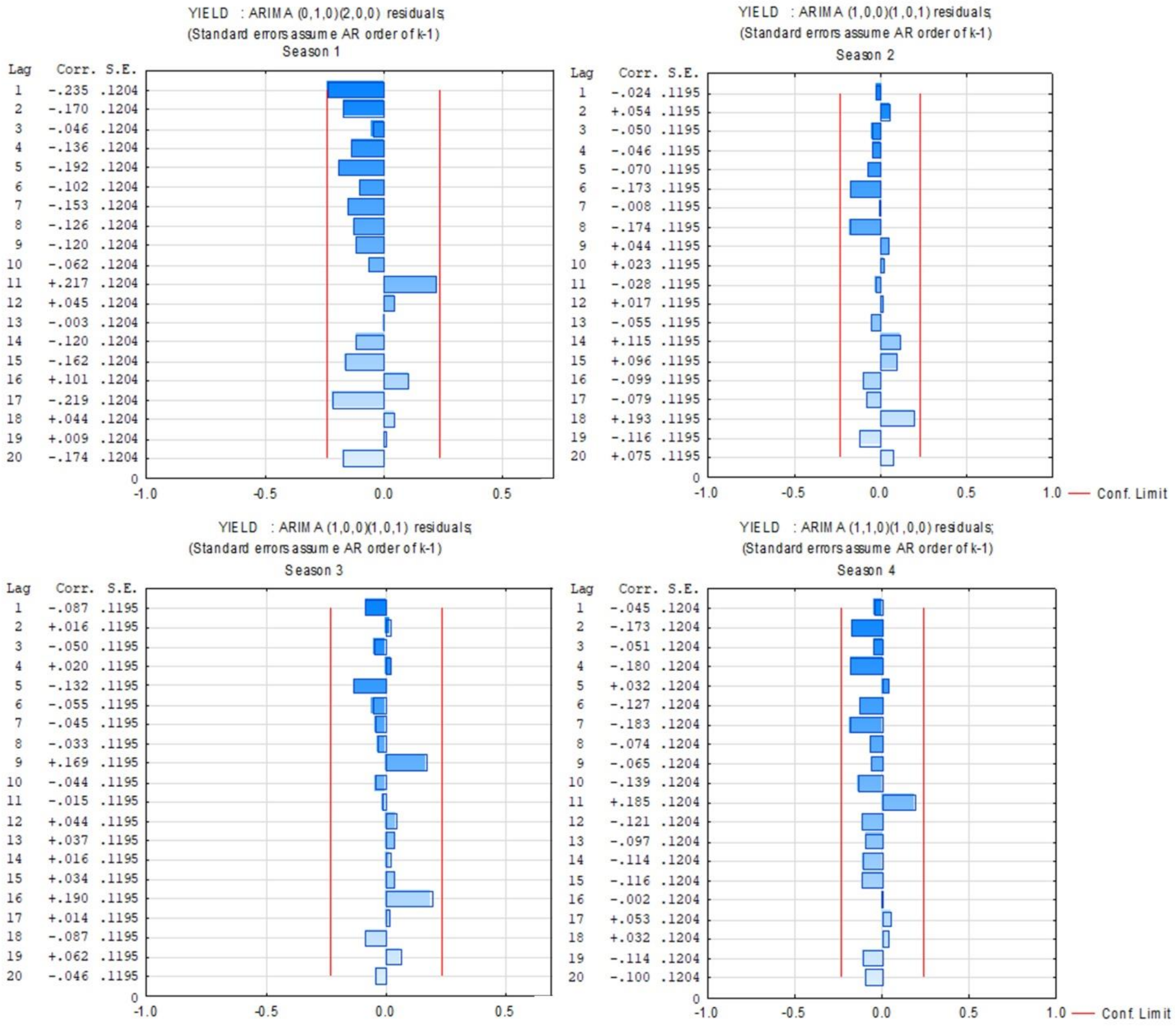

Supplementary Fig. S2A. The correlograms of the partial autocorrelations for the best selected SARIMA models for the younger primiparae (at the age of 20 – 26 months)

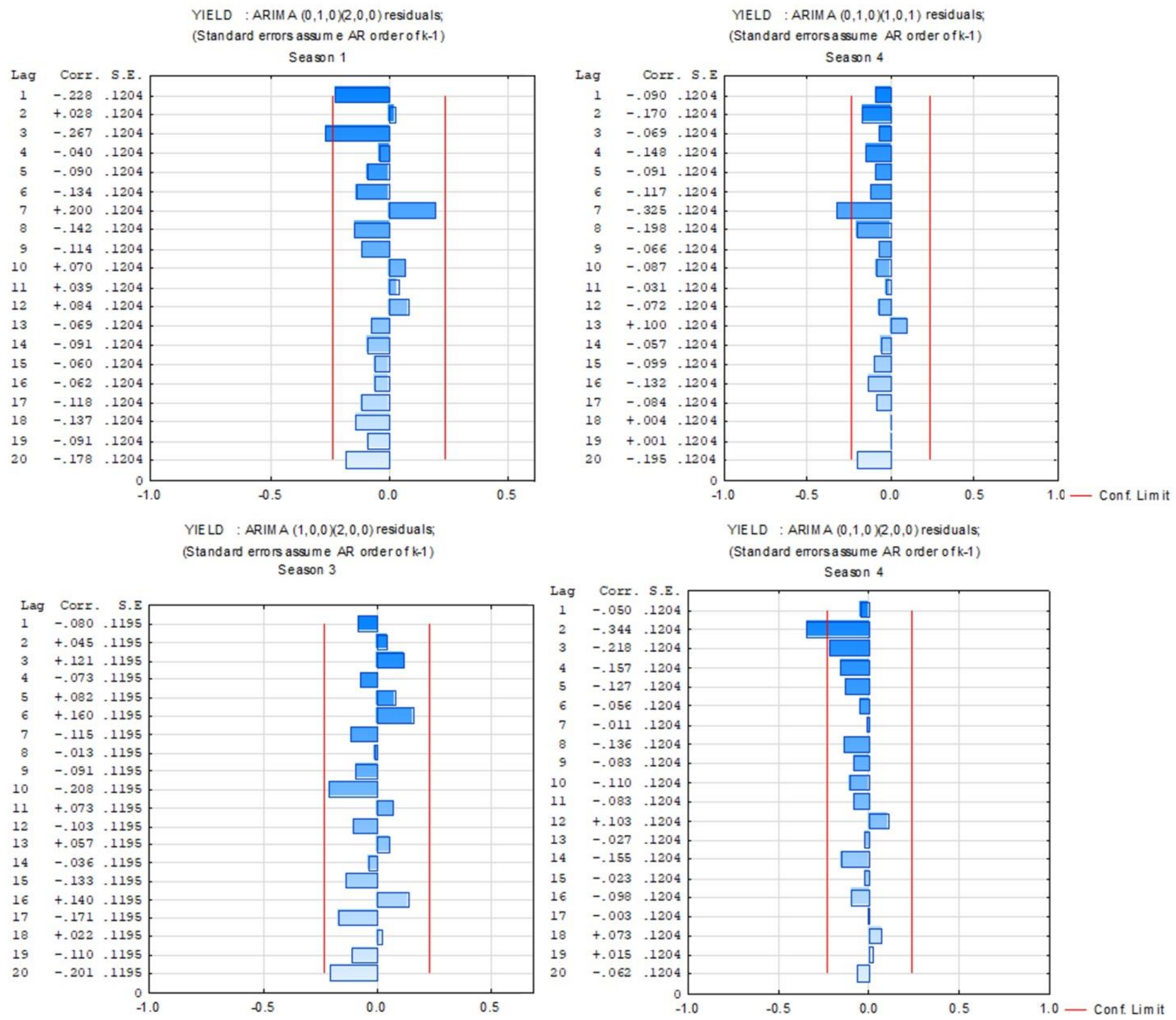

Supplementary Fig. S2B. The correlograms of the partial autocorrelations for the best selected SARIMA models for the older primiparae (at the age of 27 – 32 months)
